# The Efficacy and Safety of Intrathecal Autologous Bone Marrow-Derived Mesenchymal Stromal Cells in Amyotrophic Lateral Sclerosis: A Pilot Study

**DOI:** 10.34172/apb.2023.043

**Published:** 2022-01-08

**Authors:** Gholamreza Shamsaei, Fatemeh Houshmand, Ahmad Ahmadzadeh Deylami, Armita Valizadeh, Shahram Rafie, Maryam Moradi

**Affiliations:** ^1^Department of Neurology, Faculty of Medicine, Ahvaz Jundishapur University of Medical Sciences, Ahvaz, Iran.; ^2^Department of Hematology, Faculty of Medicine, Ahvaz Jundishapur University of Medical Sciences, Ahvaz, Iran.; ^3^Department of Anatomy, Faculty of Medicine, Ahvaz Jundishapur University of Medical Sciences, Ahvaz, Iran.; ^4^Department of Biostatistics and Epidemiology, School of Public Health, Ahvaz Jundishapur University of Medical Sciences, Ahvaz, Iran.

**Keywords:** ALS, MSCs, Mesenchymal stromal cells, Amyotrophic lateral sclerosis, Transplantation

## Abstract

***Purpose:*** Amyotrophic lateral sclerosis (ALS) is an uncommon and aggressive neurodegenerative disorder that influences the lower and upper motor neurons. There are low eligible drugs for ALS treatment; in this regard, supplemental and replacement treatments are essential. There are relative studies in the field of mesenchymal stromal cells (MSCs) therapy in ALS, but the different methods, differently used medium, and difference in follow-up periods affect the outcome treatment.

***Methods:*** The current survey is a single-center, phase I clinical trial to evaluating the efficacy and safety of autologous bone marrow (BM)-derived MSCs through intrathecal administration in ALS patients. MNCs were isolated from BM specimens and cultured. The clinical outcome was evaluated based Revised Amyotrophic Lateral Sclerosis Functional Rating (ALSFRS-R) Scale.

***Results:*** Each patient received 15±3×10^6^ cells through subarachnoid space. No adverse events (AEs) were detected. Just one patient experienced a mild headache after injection. Following injection, no new intradural cerebrospinal pathology transplant-related was observed. None of the patients’ pathologic disruptions following transplantation were detected by magnetic resonance imaging (MRI). The additional analyses have shown the average rate of ALSFRS-R score and forced vital capacity (FVC) reduction have decreased during 10 months following MSCs transplantation versus the pretreatment period, from -5.4±2.3 to -2±3.08 ALSFRS-R points/period (*P*=0.014) and -12.6±5.22% to -4.8±14.72%/period (*P*<0.001), respectively.

***Conclusion:*** These results have shown that autologous MSCs transplantation reduces the disease’s progression and has favorable safety.

***Trial Registration:*** This study performed as a phase I clinical trial (code IRCT20200828048551N1).

## Introduction

 Amyotrophic lateral sclerosis (ALS) is an uncommon and aggressive neurodegenerative disorder that influences the lower and upper motor neurons.^1,2^ Among the motor neurons disorders, ALS is more common. There are two types of ALS: hereditary and sporadic; that the sporadic form is more prevalent. In the hereditary form which is accounts for 10% of patients; asexual chromosomes are the most common pattern of inheritance.^3^ Although the main pathogenesis of the disease is not definitely recognized known, nevertheless the contribution of autoimmune mechanisms cannot be ruled out.^4^ The prevalence of ALS has been estimated at 6 cases per 100 000.^5^ The occurrence of ALS with increasing age increases, and most of the cases are 60 years or older.^5,6^ In the case of sporadic ALS, this age range increases.

 The spectrum of symptoms differs from muscle weakness to cognitive and behavioral signs, which can cause misdiagnosis.^2,7^ In accordance with clinical symptoms, ALS is categorized into two groups; spinal ALS and Bulbar ALS. In spinal ALS, patients become paralyzed or die within three to five years. The median survival duration after diagnosis, approximately 2-4 years, was reported.^8^ Despite, the recognizing prognosis factors, just 5% of patients live for 20 years or longer.^9^ Factors such as the age of onset symptoms at older age, lower Amyotrophic Lateral Sclerosis Functional Rating Scale (ALSFRS or ALSFRS-R) score, and early dysfunction of the respiratory muscles accompanied with lower survival have to influence on disease progression.^9^ Also, men are more susceptible than women to ALS.^10^

 There is no definitive treatment for ALS. Up to now, due to invasive and fast disease progression, the current therapy approach is to reduce the severity of symptoms. In this regard, more recent attention has focused on providing treatment with considerable safety and efficacy. This causes a growing body of kinds of literature focusing on the different disorder pros.^11-15^ Despite progress in understanding the therapeutic target of ALS, but there is no data as a clinical trial.^16^ Although safety treatments have developed, but effective treatment has not yet been achieved.^17^ Cell therapy in neurology disorder has been widely used. Therapeutic effects of cell therapy have been shown in the treatment of neurology disorders such as Parkinson, Alzheimer, multiple sclerosis, stroke, and Huntington.^18-20^ The literature has recently emphasized the importance of mesenchymal stromal cells (MSCs) therapeutic effect in ALS. Although MSCs have less ability to proliferate and differentiate than embryonic stem cells and neuronal stem cells, because they are autologous, they have attracted a lot of attention and have been effective in treating a number of neurological diseases. In autologous MSCs therapy, the neuroprotection of MSCs protects the surviving motor neurons.^2^

 There are relative studies in the field of MSCs therapy in ALS, but the different methods, differently used medium, and difference in follow-up periods affect the treatment progression and outcome. Hence, more investigations are needed to validate this approach. The current survey aims to evaluate the safety and efficacy of autologous bone marrow (BM)-derived MSCs injection through intrathecal in ALS patients.

## Methods

###  Study design 

 The current survey is a single-center, phase I clinical trial to evaluating the efficacy and safety of autologous BM-derived MSCs through intrathecal administration in ALS patients were recruited to the department of neurology. The study was based on the approval of the Medical Ethics Committee of Jundishapur Ahvaz University (Reference Number: IR.AJUMS.REC.1398.904) and performed as a phase I clinical trial (code IRCT20200828048551N1). All participants assigned the consent form.

###  Study population

 Selecting patients for the current study were based on the following inclusion criteria: age between 20 and 60 years old, the Amyotrophic Lateral Sclerosis Functional Rating Scale-Revised (ALSFRS-R) score between 26 and 46, forced vital capacity (FVC) of more than 40% of the predicted value, and history of at least 3 months before screening riluzole treatment at the stable dose (50 mg, twice daily). Exclusion criteria were considered as previous treatment with stem cell therapy, pregnancy, breastfeeding, coagulopathy, gastrostomy, cardiovascular disease, hepatic or renal disorder, cancer, systemic infection, recurrent thromboembolic disease, tracheostomy ventilation, or noninvasive ventilation (NIV) for more than 12 hours per day, and the administration of any drug which influences the BM. All patients are assigned a consent form.

###  BM-derived mesenchymal stromal cells production

 BM aspiration was performed under short general anesthesia with a puncture from the posterior superior iliac crest while the patient was lying in a left or a right lateral position. Approximately 200 mL of BM inocula was obtained from each patient.

 Mononuclear cells (MNCs) were isolated from BM specimens; at first, the samples were diluted (1:1) with phosphate-buffered saline (PBS, pH 7.4, Gibco, BRL) next separated by using Ficoll (Biosera, France) and density gradient centrifuge (1500 rpm for 20 minutes). Then MNCs were cultured under standard conditions in a DMEM-F12 medium containing human serum (15%) and antibiotics in a 25 cm flask (day 0). The flasks were incubated in 5% CO2 and 37°C. To removing the non-adherent cells were washed using PBS. This process was repeated until the cells reached 80-90% confluent, then MSCs were harvested using trypsin enzyme (Gibco, BRL) and subcultured. The MSCs were analyzed with flow cytometry for markers and features described in International Society for Cellular Therapy guidelines.^21^

###  Cell transplantation

 Each patient received 15 ± 3 × 10^6^ cells through subarachnoid space using lumbar puncture at L2-3 or L3-4 levels. After transplantation, patients were closely monitored within 1 day for immediate adverse events (AE). The critical clinical findings, including respiratory and heart rate, blood pressure, and body temperature, were assessed every 6 hours. After 24 hours, if any AEs were not observed, patients were discharged.

###  Clinical assessment

 The clinical outcome was evaluated based on ALSFRS-R.^22^ Respiratory failures account for one of the fatal complications of ALS. In this regard, in the present survey, respiratory function is evaluated by FVC routinely.^23,24^ To assess MSCs therapy safety, patients were monitored for AEs, including liver, kidney, and thyroid function, for 20 months (-10 months to + 10 months) before transplantation conditions of all patients were evaluated. After transplantation, all patients were evaluated for AEs by magnetic resonance imaging (MRI).

###  Statistical analysis 

 Normality of data were checked by Kolmogorov-Smirnov test. T-test was used to compare baseline parameters after follow-up. The ANOVA test was used to compare the outcome of transplantation with time. Mann-Whitney was used to compare means. The significant P-value was considered < 0.05. All data were analyzed by SPSS software (V24, 2017, USA).

## Results and Discussion

 Seven patients (5 males and 2 females) were recruited for this study. The mean age was 44.7 ± 9.01 years. All patients were diagnosed according to the El Escorial revised criteria.^25^ Two patients underwent a tracheostomy due to progressive respiratory failure before treatment and were excluded from the study. The baseline characteristics and functional changes of the initial 7 patients are described in Table 1. Five patients who matched the selection criteria were followed up. At -10 months, the mean of ALSFRS-R score and FVC were 32.2 ± 5.58 points and 55.2% ± 19.18, respectively. For safety evaluation, all patients were assessed regulatory during the following-up period. No AEs were detected in patients (Table 2). Just one patient experienced a mild headache after injection, which resolved with routine analgesics. Following injection, no new intradural cerebrospinal pathology transplant-related was observed. None of the patients’ pathologic disruptions following transplantation were not detected in patients by MRI. Two patients could not complete the follow-up period due to experiencing tracheostomy causes progressive respiratory failure and the efficacy was calculated for five patients. The site and route injection are important factors that can influence the safety and efficacy of treatment. Nabavi and colleagues carried out a clinical trial to compare the safety and efficacy of intravenous and intrathecal MSCs injections; they demonstrated without significant AEs, both injections are helpful in ALS cell therapy.^26^ Also, it was demonstrated in patients with widespread tissue damage, MSCs injection in different sites has the safety and efficacy with injection through the spinal cord.^27^ Hence, in the current investigation, the MSCs were administrated in a noninvasive way. Our findings have shown the single dose of MSCs injection through the intrathecal route without any life-threatening AEs increase the ALSFRS-R slope. This result is in accordance with Berry and colleagues and Syková and colleague’s results; they reported a single dose of MSCs injection to improve the ALSFRS-R slope than the placebo group.^28,29^

**Table 1:** The clinical characteristic of patients at baseline

**Patients**	**Age (y)**	**Gender**	**Disease duration**^b^	**Disorder Type**^b^	**ALSFRS-R score**	**FVC (%)**	**PEG**^a^	**Tracheostomy**^a^	**Death**^a^
1	35	M	30	Spinal	30	55	N0	NO	NO
2	53	M	14	Spinal	44	96	NO	NO	NO
3	44	F	24	Spinal + bulbar	29	42	NO	NO	NO
4	53	M	22	Bulbar	34	59	NO	NO	NO
5	32	M	20	Spinal	31	49	NO	NO	NO
6*	42	F	18	Bulbar	31	46	-2	-2	NO
7*	54	M	34	Spinal	27	40	-1	-1	NO

Abbreviations: ALS: amyotrophic lateral sclerosis; ALSFRS-R: Revised ALS Functional Rating Scale; PEG: percutaneous endoscopic gastrostomy *Patients 6 and 7 were excluded because of insufficient long-term follow-up data (for the reasons of tracheostomy). ^a^ Month from injection; ^b^ at enrollment (mon).

**Table 2:** Adverse effects after cell transplantation

**Adverse effect**	**Patient (n)**	**Duration (days)**	**Outcome**
General disorders and administration site condition			
Influenza-like illness	0	No	-
Pyrexia	0	No	-
Pain	0	No	-
Nervous system disorders			
Headache	1	5	Improved after treatment
Nausea and vomiting	0	No	-
Dizziness	0	No	-
Unconsciousness	0	No	-
Seizures	0	No	-
Vertigo	0	No	-
Visual impairment	0	No	-
Musculoskeletal and connective tissue disorders			
Back pain	0	No	-
Arthralgia	0	No	-
General disorders administration site condition			
Influenza-like illness	0	No	-
Pyrexia	0	No	-
Allergic reactions			
Fever	0	No	-
Anaphylaxis	0	No	-
Apnea	0	No	-
Urticaria	0	No	-
Local adverse events			
Infection	0	No	-
Phlebitis	0	No	-
Hematoma	0	No	-

**Figure 1:**
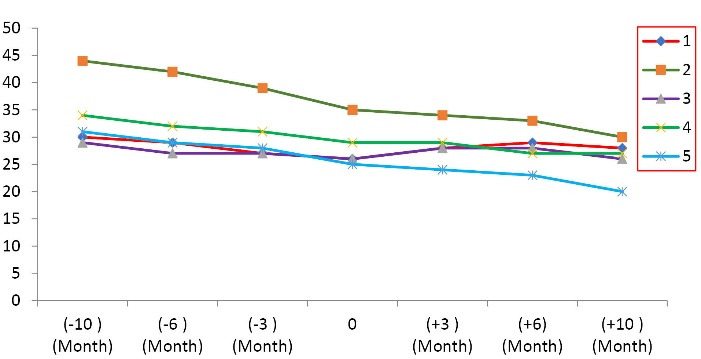
The trend of the amyotrophic lateral sclerosis-functional rating scale (ALS-FRS) during 10 months follow-up.

**Figure 2:**
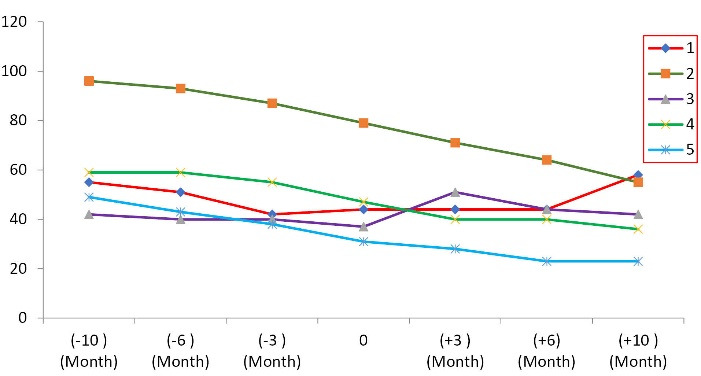
The trend of forced vital capacity (FVC) during 10 months follow-up.

 The additional analyses have shown the average rate of reduction of ALSFRS-R score and FVC have decreased during 10 months following MSCs transplantation versus the pretreatment period, from -5.4 ± 2.3 to -2 ± 3.08 ALSFRS-R points/period (*P* = 0.014) (Table 3, Figure 1) and -12.6 ± 5.22 % to -4.8 ± 14.72 %/period (Figure 2, Table 4) (*P* < 0.001), respectively. These results indicate that the autologous MSCs transplantation reduces the disease progression. Further analysis with linear regression has revealed that 43% of changes after transplantation have belonged to FVC, and this value for ALSFRS-R score was detected 65.7% (Table 5). The effect size of autologous MSCs transplantation was shown in Table 5.

**Table 3:** ALSFRS-R score of all 5 ALS patients enrolled in the clinical trial

**Patient**	**-10**	**-6**	**-3**	**0**	**3**	**6**	**10**
	**M**	**M**	**M**	**M**	**M**	**M**	**M**
1	30	29	27	26	28	29	28
2	44	42	39	35	34	33	30
3	29	27	27	26	28	28	26
4	34	32	31	29	29	27	27
5	31	29	28	25	24	23	20

* The negative symbol (-) before month indicating the period of pretransplant.

**Table 4:** Forced vital capacity of All 5 ALS Patients Enrolled in the Clinical trial

**Patient**	**-10**	**-6**	**-3**	**0**	**3**	**6**	**10**
	**M**	**M**	**M**	**M**	**M**	**M**	**M**
1	55	51	42	44	44	44	58
2	96	93	87	79	71	64	55
3	42	40	40	37	51	44	42
4	59	59	55	47	40	40	36
5	49	43	38	31	28	23	23

**Table 5:** The regression analysis and effect size for FVC and ALSFRS-R score

	**Group 1 (SD)**	**Group 2 (SD)**	**Effect Size Cohen's d (95% CI)**	**R**	**R**^2^	**Adjusted R**^2^
FVC	12.6 (5.22)	4.8 (14.72)	-0.706	0.659	0.434	0.245
ALSFRS-R score	5.4 (2.30)	2 (3.08)	-1.251	0.810	0.657	0.542

Abbreviations: ALSFRS-R: Revised ALS Functional Rating Scale; FVC: forced vital capacity; CI: confidence interval; MSCs: mesenchymal stromal cells; SD: standard deviation.

 The Pearson correlation coefficients were used to determine the association between ALSFRS-R score and FVC parameters before and after treatment. However, the significant differences were not seen (*P* = 0.096 and *P* = 0.227), respectively.

 More recent attention has focused on providing usability of MSCs therapy in neurodegenerative disorders due to their unique abilities. There are some comparative studies conducted on the efficacy of MSCs therapy in ALS. However, the determination type of administration route, count of injecting cells, and several injections are technically challenging, so there is a need for more investigations in line with similar previous surveys. The present study was designed to determine the therapeutic safety and efficacy of intrathecal injection of autologous MSCs on ALS progress and outcome.

 Neuroinflammation plays a key role in ALS pathogenesis. The imbalance between inflammatory and anti-inflammatory is one of the main pathogenesis in ALS. Analysis of the involved cytokines and chemokines in ALS revealed CCL2, CXCL8, CCL4, and Transforming growth factor-β (TGF-β) have negatively correlated with ALSFRS-R score and disease progression.^23,30^ In this regard, it was demonstrated MSCs, by increasing TGF-β secreting, disrupt the motor neuron loss in ALS.^31^ So, the therapeutic strategy to balance inflammatory and anti-inflammatory cytokines causes a growing body of literature in this issue.

 TAR DNA-binding protein 43 (TDP-43) is a protein which normally located in nuclei cells. In a considerable number of ALS cases, the TDP-43 in insoluble shortened form accumulates in the cell cytoplasm.^32^ In the hereditary form of ALS, the mutation in the C9otf72 gene causes neurodegenerative.^33^ This gene defect with influencing TDP-43 protein contributes to ALS pathogenesis.^34,35^ These aggregations change the shape of the membrane and the nucleus pores, thereby disrupting the entry of important proteins into the nucleus and the exit of RNA from the nucleus. Additionally, these accumulations with upregulation of nuclear factor кB (NF-κB) and type I interferon (IFN) pathways and with involving neuroinflammation contributes to ALS pathogenesis.^36^

 Monocyte chemoattractant protein-1 (MCP-1) by disrupting motor neurons contribute to ALS pathogenesis.^37^ By decreasing MCP-1, the severity of ALS symptoms is reduced; subsequently, the ALSFR-R score decreases. MSCs by decreasing MCP in CSF-1 and stromal cell-derived factor-1a (SDF-1) after transplantation and effect on ALSFR-S score.^28^ inflammatory cytokines by increasing MCP-1 Intensifies and causes fast progression in ALS patients^38^; decreases the average rate of reduction of ALSFRS-R score and FVC after single-dose MSCs injection in our result can be explained by this and confirms that by repeated injection, the number of cells increases in CSF for a longer time and can be more therapeutic than single-dose.^39^

 In a study that determined whom ALS patients respond better to autologous MSCs transplantation, Kim and colleagues isolated MSCs from responder and non-responder and induction to mice “to measure their lifespans locomotor activity, and motor neuron numbers.” Their findings have shown that TGF-β, vascular endothelial growth factor, and angiogenin are higher in responders than non-responders; in fact, these cytokines can be used as a predictor for responding to the treatment and can be helpful to in decision whom patients need repeated injection.^40^ This finding is worth it and can justify the result of Siwek and colleagues that after repeated administration of MSCs, the favorable outcome was not observed.^1^

 MSCs, by reducing neuronal sensitivity to glutamate receptor ligands and altering gene expression, have a neuroprotection effect on damaged CNS.^41^ The absence of any AEs and no sign of abnormal cell growth after transplantation indicates that our intervention is safe and successfully improved ALS treatment.

## Conclusion

 The most prominent finding to emerge from this study is like the previous surveys that MSCs transplantation has favorable safety. In the current trial also demonstrated that MSCs auto transplantation is accompanied by slow disease progression in some ALS patients compare pretreatment period, indicating the efficacy of treatment. Unfortunately, the study did not include the cytokines and inflammation biomarkers before and after treatment. Further studies with repeated injection and evaluation predicting more biomarkers to therapy with a larger sample size are strongly suggested.

## Acknowledgments

 We wish to thank all our colleagues in Allied Health Sciences School, Ahvaz University of medical sciences.

## Competing Interests

 The authors declare no conflict of interest. All procedure performs in studies involving human participants were in accordance with the ethical standards of the institutional and/or national research committee and with the 1964 Helsinki Declaration and its later amendments or compare ethical strand.

## Ethical Approval

 The study was based on the approval of the Medical Ethics Committee of Jundishapur Ahvaz University (Reference Number: IR.AJUMS.REC.1398.904) and performed as a phase I clinical trial (identifier: IRCT20200828048551N1). All participants assigned the consent form.
